# Corneal Langerhans cells in children with celiac disease

**DOI:** 10.1038/s41598-022-22376-w

**Published:** 2022-10-31

**Authors:** Hoda Gad, Ibrahim Mohammed, Saras Saraswathi, Bara Al-Jarrah, Maryam Ferdousi, Ioannis N. Petropoulos, Georgios Ponirakis, Adnan Khan, Parul Singh, Souhaila Al Khodor, Mamoun Elawad, Wesam Almasri, Hatim Abdelrahman, Khalid Hussain, Mohamed A. Hendaus, Fatma Al-Mudahka, Khaled Abouhazima, Anthony K. Akobeng, Rayaz A. Malik

**Affiliations:** 1 Department Medicine, Weill Cornell Medicine-Qatar, Doha, Qatar; 2 Department of Internal Medicine, Albany Medical Center Hospital, Albany, NY USA; 3 Division of Gastroenterology, Hepatology, and Nutrition, Sidra Medicine, Doha, Qatar; 4 Institute of Cardiovascular Medicine, University of Manchester, Manchester, UK; 5 Faculty of Health Sciences, Khyber Medical University, Peshawar, Pakistan; 6 Research Department, Sidra Medicine, Doha, Qatar; 7 Division of Endocrinology, Sidra Medicine, Doha, Qatar; 8 General Pediatrics Department, Sidra Medicine, Doha, Qatar

**Keywords:** Peripheral nervous system, Gastroenterology, Neurology

## Abstract

Celiac disease (CeD) is a common small bowel enteropathy characterized by an altered adaptive immune system and increased mucosal antigen presenting cells. This study aims to establish if quantification of corneal Langerhans cells (LCs) using corneal confocal microscopy (CCM) could act as a surrogate marker for antigen presenting cell status and hence disease activity in children with CeD. Twenty children with stable CeD and 20 age-matched controls underwent CCM and quantification of central corneal total, mature and immature LC density. There was no difference in age (11.78 ± 1.7 vs. 12.83 ± 1.91; *P* = 0.077) or height (1.38 ± 0.14 vs. 1.44 ± 0.13; *P* = 0.125). BMI (18.81 ± 3.90 vs. 22.26 ± 5.47; *P* = 0.031) and 25 OHD levels (43.50 ± 13.36 vs. 59.77 ± 22.45; *P* = 0.014) were significantly lower in children with CeD compared to controls. The total (33.33(16.67–59.37) vs. 51.56(30.21–85.42); *P* = 0.343), immature (33.33(16.67–52.08) vs. 44.79(29.17–82.29); *P* = 0.752) and mature (1.56(0–5) vs. 1.56(1.04–8.33); *P* = 0.752) LC density did not differ between the CeD and control groups. However, immature (r = 0.535, *P* = 0.015), mature (r = 0.464, *P* = 0.039), and total (r = 0.548, *P* = 0.012) LC density correlated with age. Immature (r = 0.602, *P* = 0.038) and total (r = 0.637, *P* = 0.026) LC density also correlated with tissue transglutaminase antibody (Anti-TtG) levels assessed in 12/20 subjects with CeD. There was no difference in corneal LC density between children with CeD and controls. However, the correlation between corneal LC density and anti-TtG levels suggests a relationship with disease activity in CeD and requires further study.

## Introduction

Celiac disease (CeD) affects ~ 0.7% of the world population^1^, but may be more prevalent in the Middle East^2,3^, especially in Qatar^4^. It is characterized by varying degrees of intestinal malabsorption, caused by an inappropriate immune response to ingested wheat gluten containing gliadin. Histopathological studies demonstrate villous atrophy with defective transepithelial^5,6^ and paracellular uptake of gliadin by the intestinal mucosa of patients with active celiac disease.

Circulating dendritic cells are recruited to the inflamed mucosa in those with active CeD, and indeed , there is a significant increase in the number of dendritic cells (DC) in the lamina propria of patients with active celiac disease, which reverts to normal with a gluten-free diet^7,8^. DCs isolated from patients with active celiac disease behave as APCs and transcribe IFN-gamma^9^, a key cytokine in the pathogenesis of CeD. The level of auto-antibodies to the enzyme transglutaminase 2 (TG2) and gliadin in gluten-consuming subjects are used as a diagnostic adjunct and marker of disease activity. Intriguingly, TG2 is expressed on most cell surfaces including monocytes and APCs, suggesting that it may facilitate the uptake of gluten^10^.

Corneal Langerhans cells (LC’s) are APCs which modulate the immune response in the cornea^11,12^. We have used corneal confocal microscopy (CCM) a rapid, non-invasive and well-tolerated ophthalmic imaging technique to quantify the number of mature and immature corneal LC’s^11–15^. Moreover, we and others have shown increased LC’s in patients with type 1 diabetes^11,16^, latent autoimmune diabetes of adults (LADA)^16^, multiple sclerosis (MS)^12^, long-COVID^17^, dry eye disease^18^, systemic lupus erythematosus (SLE)^19^, fibromyalgia^20^, thyroid-associated ophthalmopathy^21^, and chronic inflammatory demyelinating polyneuropathy (CIDP)^22^. These studies suggest that corneal immune cells are associated with a number of immune and inflammatory diseases. In the present study we have quantified mature, immature and total numbers of LCs and related them to clinical parameters and anti-TtG level in children with CeD.

## Results

There was no significant difference in age, height, or tissue transglutaminase antibody between children with CeD and the control group. BMI and vitamin D were significantly lower in children with CeD compared to the control group (Table 1).

**Table 1:** Clinical demographic and laboratory measures in control subjects and children with CeD.

	Controls (n = 20)	CeD (n = 20)	*P*-value
Age	12.83 ± 1.91	11.78 ± 1.74	0.077
Duration of disease	-	4.49 ± 4.02	–
Height (m)	1.44 ± 0.13	1.38 ± 0.14	0.125
BMI (kg/m^2^)	22.26 ± 5.47	18.81 ± 3.90	**0.031**
Tissue Transglutaminase antibody (U/mL)	0–3	12.90(1–165)*	–
Hemoglobin (g/dl)	125.50 ± 7.84	125.63 ± 7.45	0.963
Platelets × 10^^9^/L	333.75 ± 73.20	325.42 ± 77.51	0.768
WBC × 10^^9^/L	7.72 ± 1.98	6.73 ± 1.89	0.171
25 OHD (ng/mL)	59.77 ± 22.45	43.50 ± 13.36	**0.014**
Vitamin B_12_ (ng/mL)	180–914	321.84 ± 89.40	–
Folic acid (nmol/L)	11.3–47.6	18.60(12.20–45.50)	–
Serum Iron (μmol/L)	13.65 ± 2.76	11.46 ± 7.67	0.700

Data is presented as mean ± SD or median (range), Anti TtG, vitamin B12 and folic acid levels were compared with the normal laboratory range. CeD: Celiac Disease, BMI: Body Mass Index, WBC: White blood Cell Count. *Anti-TtG was available in 12/20 children with CD. Significant differences between groups are highlighted in bold.

### Langerhans cells

**Figure 1:**
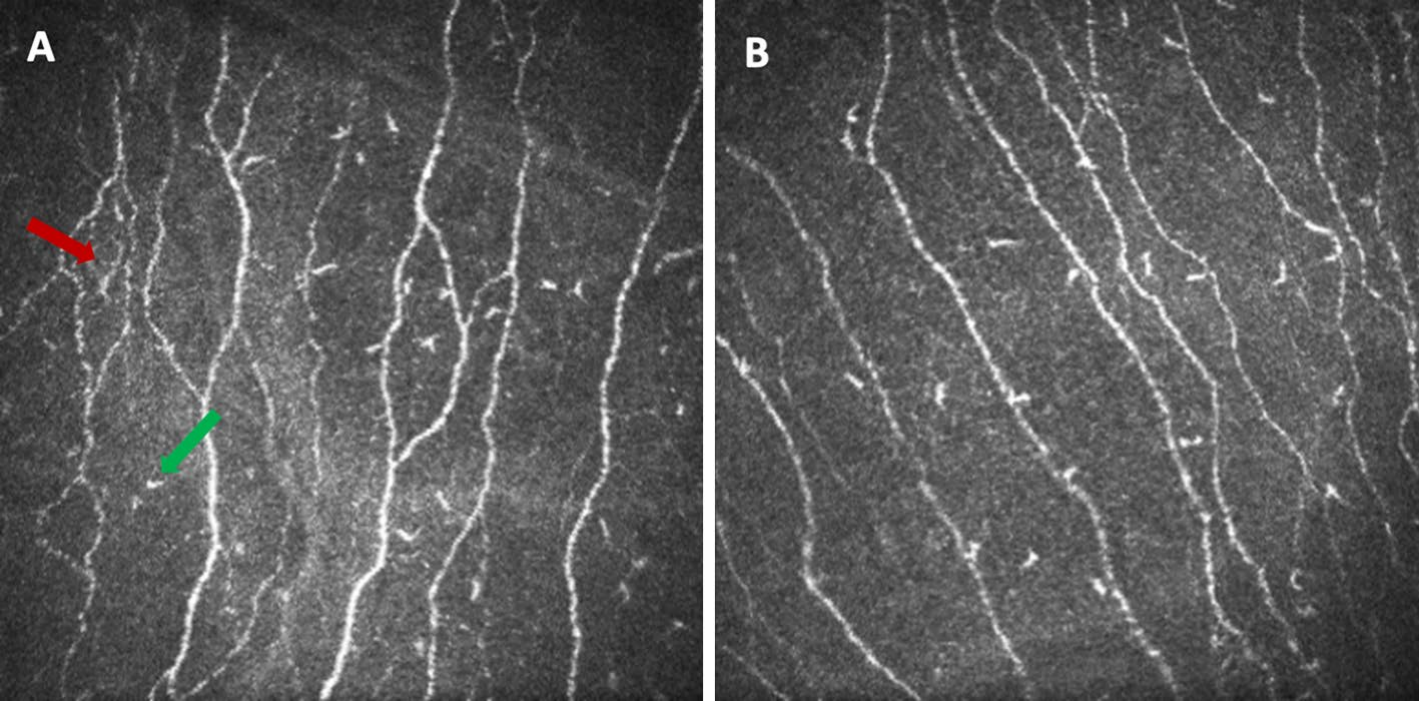
(**a**) A CCM image of the sub-basal nerve plexus in a healthy control and (**b**) a child with celiac disease (CeD) with mature (red arrow) and immature LCs (green arrow).

The total, immature and mature LC density in the central cornea did not differ between children with CeD and controls (Fig. 1a,b) (Table 2).

**Table 2:** Langerhans cells in children with Celiac Disease and controls.

Density (no./mm^2^)	Controls	Celiac Disease	*P*-value
Immature LC	44.79(29.17–82.29)	33.33(16.67–52.08)	0.752
Mature LC	1.56(1.04–8.33)	1.56(0–5)	0.752
Total LC	51.56(30.21–85.42)	33.33(16.67–59.37)	0.343

*Data is presented as mean ± SD, CeD: celiac disease, LC: Langerhans cells.

### Correlation between Langerhans cell densities and clinical parameters.

**Figure 2:**
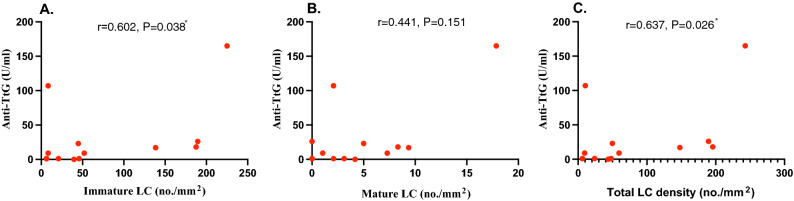
Correlation between Anti-TtG levels and immature (**a**), mature (**b**) and (**c**) total LC density in children with stable CeD.

Using spearman correlation, immature (r = 0.535, *P* = 0.015), mature (r = 0.464, *P* = 0.039), and total (r = 0.548, *P* = 0.012) LC density correlated with age. In 12/20 children with CeD who had recently undergone assessment of anti-TtG levels, there was a significant correlation with immature LC density (r = 0.602, *P* = 0.038) and total LC density (r = 0.637, *P* = 0.026) (Fig. 2a–c).

## Discussion

Gluten-induced activation of the adaptive immune response and immune mediated tissue inflammation occurs in celiac disease. We show normal anti-TtG levels and LC density in children with stable CeD. Previous studies have shown increased LC’s in a variety of autoimmune conditions including diabetes^11,16^, latent autoimmune diabetes of adults (LADA)^16^, multiple sclerosis (MS)^12^, long-COVID^17^, dry eye disease^18^, systemic lupus erythematous (SLE)^19^ and fibromyalgia^20^. Our group was the the first to show increased LC’s in patients with chronic inflammatory demyelinating polyneuropathy (CIDP), suggesting that corneal LC’s may be a surrogate marker of antigen presenting cell status in peripheral nerves^22^. Indeed, recent studies in patients with CIDP have shown that increased LC’s may predict increased rates of disease progression and neurological disability^23,24^. Furthermore, treatment of a 27-year-old patient with subacute anti-neurofascin-155 neuropathy with rituximab was associated with an improvement in clinical and neurophysiological parameters and a reduction in antibody titers and LC density^25^.

Interestingly, despite overall normal anti-TtG level, immature and total LC densities correlated with anti-TtG levels. Anti-TtG levels are increased in CeD, but also other inflammatory conditions such as Crohn’s disease, ulcerative colitis, primary biliary cirrhosis^26^, rheumatoid arthritis^27^, and chronic liver disease^28^ which has been attributed to an overexpression of TtG, and its involvement in apoptosis, tissue repair, and fibrosis^29^.

We also found a lower level of 25OHD in patients with CeD, consistent with a higher prevalence of vitamin D deficiency in children with newly diagnosed celiac disease^30^ and a non-linear relationship between vitamin D deficiency and the development of CeD in at-risk children^31^. In a recent study of 200 Saudi adolescent girls with marked vitamin D deficiency a strong inverse correlation was observed between TtG and 25 OHD levels^32^ . Vitamin D is a key mediator of innate immune function and signals directly to alter macrophage and dendritic cell function^33^. We found no correlation between 25OHD levels and LC density, although a previous study demonstrated an inverse correlation between vitamin D levels and dendritic cells in patients with dry eye^34^.

In conclusion, children with stable CeD have normal corneal LC numbers. However, even though, anti-TtG levels were within the normal range there was a correlation with LC density, suggesting there may be sub-clinical immune activation in children with stable CeD. This is an exploratory study with a cross-sectional design and small sample size. Nevertheless, the association between anti-TtG levels and LC’s merits further study to assess if corneal LCs could be used to monitor disease activity and adherence to a gluten-free diet in patients with CeD.

## Methods

Children with a confirmed diagnosis of celiac disease and a positive serology test for anti-TtG antibodies (available in 12/20) with a disease duration of 4.49 ± 4.02 years and 20 healthy controls were recruited. Inclusion criteria were age between 8 and 17 years and a diagnosis of CeD. Exclusion criteria were any history of a cause of neuropathy, malignancy, vitamin B12 or folate deficiency, liver, or renal dysfunction. Participants were also excluded in they had corneal pathology, allergy to the eye-drops or previous ocular trauma or surgery in the past six months. Participants with a history of or current contact lens use were excluded. The study was approved by the Ethics Committee of Weill Cornell Medicine-Qatar (IRB 1700032) and Sidra Medicine (IRB 1500758–3) and was undertaken according to the principles of the Helsinki Declaration. Written informed consent and assent were obtained from all participants and their parents.

### Corneal confocal microscopy procedure

Corneal confocal microscopy was undertaken using the Heidelberg Retina Tomograph Cornea Module (Heidelberg Engineering, Germany). Anesthetic drops Bausch & Lomb Minims ® (Oxybuprocaine hydrochloride 0.4% w/v) were used to numb both eyes to limit irritation and discomfort during the examination. A drop of hypotears gel (Carbomer 0.2% eye gel) was placed on the tip of the objective lens and a sterile disposable TomoCap was placed over the lens, allowing optical coupling of the objective lens to the cornea. Images were captured from the central cornea to quantify corneal Langerhans cells in the sub-basal layer. The investigator (HG) was blind to the study group when performing CCM and analyzing CCM images.

### Image selection and quantification

Six images were selected from the central cornea, excluding those with pressure lines and out of focus images. Langerhans cells were identified as white, bright structures^13^. Total, mature (with dendrites) and immature (without dendrites and a total end-to-end length less than 25 µm)^35^ LC’s were counted manually using CCMetrics software^11^. The investigator was blind to disease and control group during selection and quantification of the CCM images using anonymized codes for each participant.

### Statistical analysis

All statistical analyses were performed using IBM SPSS Statistics software Version 27 and *P* < 0.05 was considered statistically significant. Normality of the data was assessed using the Shapiro–Wilk test, histograms and normal Q-Q plot. Data are expressed as mean ± SD for the normally distributed variables and as median(range) for the skewed variables. Inferential analyses were conducted for the corneal nerve parameters and clinical demographics using both parametric (T-test) and non-parametric (Mann–Whitney U) tests, with post-hoc adjustment. To investigate the association between corneal nerve metrics and clinical variables, Pearson and Spearman correlation were performed as appropriate. Graphpad prism version 9 was used to build dot plots.

## Data Availability

Data is available upon reasonable request to the corresponding author.
